# 3,8-Dimethyl-4,7-diaza­deca-3,7-diene-2,9-dione dioxime

**DOI:** 10.1107/S1600536810051603

**Published:** 2010-12-15

**Authors:** Supakit Achiwawanich, Tanwawan Duangtongyou, Chaveng Pakawatchai, Sutatip Siripaisarnpipat

**Affiliations:** a Center of Excellence in Functional Materials, Department of Chemistry, Faculty of Science, Kasetsart University, Bangkok 10903, Thailand; b Department of Chemistry, Faculty of Science, Prince Songkla University, Hatyai, Songkla, Thailand

## Abstract

The complete mol­ecule of the title compound, C_10_H_18_N_4_O_2_, is generated by a crystallographic inversion centre at the mid-point of the central C—C bond. The two oxime groups have an *E* configuration. In the crystal, mol­ecules are linked through inter­molecular O—H⋯N hydrogen bonds.

## Related literature

For a related synthesis and the application of the title compound as a ligand, see: Uhlig *et al.* (1966 ▶); Kitiphaisalnont *et al.* (2006 ▶).
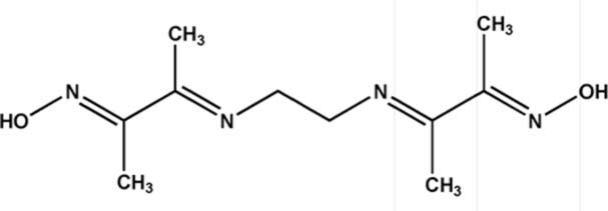


## Experimental

### 

#### Crystal data

C_10_H_18_N_4_O_2_
*M* *_r_* = 226.28
Monoclinic, 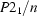

*a* = 4.4128 (3) Å
*b* = 12.8534 (8) Å
*c* = 10.4860 (7) Å
β = 90.762 (2)°
*V* = 594.71 (7) Å^3^
*Z* = 2
Mo *K*α radiation
μ = 0.09 mm^−1^
*T* = 293 K
0.16 × 0.13 × 0.12 mm

#### Data collection

Bruker SMART CCD area-detector diffractometer
3578 measured reflections
1293 independent reflections
939 reflections with *I* > 2σ(*I*)
*R*_int_ = 0.032
3 standard reflections every 120 min  intensity decay: none

#### Refinement

*R*[*F* ^2^ > 2σ(*F* ^2^)] = 0.049
*wR*(*F* ^2^) = 0.132
*S* = 1.05
1293 reflections
75 parameters
H-atom parameters constrained
Δρ_max_ = 0.16 e Å^−3^
Δρ_min_ = −0.22 e Å^−3^

### 

Data collection: *SMART* (Bruker, 2000 ▶); cell refinement: *SAINT* (Bruker, 2000 ▶); data reduction: *SAINT*; program(s) used to solve structure: *SHELXS97* (Sheldrick, 2008 ▶); program(s) used to refine structure: *SHELXL97* (Sheldrick, 2008 ▶); molecular graphics: *SHELXTL* (Sheldrick, 2008 ▶); software used to prepare material for publication: *SHELXTL*.

## Supplementary Material

Crystal structure: contains datablocks I, global. DOI: 10.1107/S1600536810051603/lx2184sup1.cif

Structure factors: contains datablocks I. DOI: 10.1107/S1600536810051603/lx2184Isup2.hkl

Additional supplementary materials:  crystallographic information; 3D view; checkCIF report

## Figures and Tables

**Table 1:** Hydrogen-bond geometry (Å, °)

*D*—H⋯*A*	*D*—H	H⋯*A*	*D*⋯*A*	*D*—H⋯*A*
O1—H1⋯N2^i^	0.82	2.12	2.8384 (19)	146

Symmetry code: (i) 
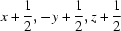
.

**Fig. 1.**
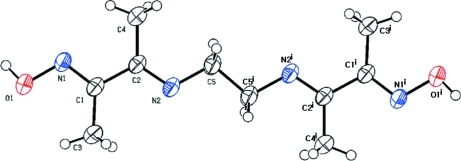
The molecular structure of the title compound with the atom numbering scheme. Displacement ellipsoids are drawn at the 50% probability level. H atoms are presented as a small spheres of arbitrary radius.[Symmetry codes:(i) -x, 1 - y, -z.]

**Fig. 2.**
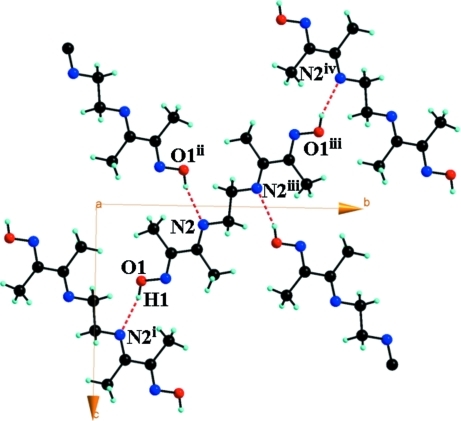
A view of O—H···N interactions (dotted lines) in the crystal structure of the title compound. [Symmetry codes: (i) 1/2 + x, 0.5 - y, 1/2 + z; (ii) -1/2 + x, 0.5 - y, -1/2 + z; (iii) -x, 1 - y, -z; (iv) -0.5 - x, 1/2 + y, -0.5 - z.]

## supplementary crystallographic information

### Comment

The diiminedioxime have been used as ligand for the complexation of transition metal ions (Uhlig *et al.*, 1966). The title molecule, *C*_10_H_18_N_4_O_2_,is a diiminedioxime which contains shortest methylene backbone. The hydrogen bonds are formed between two oxime groups (Kitiphaisalnont *et al.*, 2006). Herein, we report on the crystal structue of the title compound.

The centrosymmetric unit of the title compound (Fig. 1) is generated by a crystallographic inversion centre (symmetry code: -*x*,-*y*,-*z*) at the mid-point of the the central C—C bond and there is a crystallographic twofold screw axis (symmetry code: -*x* + 1/2,*y* + 1/2,-*z* + 1/2). The two oxime groups have a E configuration. The crystal packing (Fig. 2) is stabilized by weak intermolecular O—H···N hydrogen bonds (Table 1; O1—H1···N2^ii^).

### Experimental

The title compound was prepared from the simple reaction between diacetylmonoxime 20.2 g (0.200 mole)and ethylenediamine 6.7 ml (0.100 mole)in 50 ml me thanol. After the mixture was stirred at room temperature for 30 min, white solid precipitated (yield 75%).After recrystalization,the colorless crystals were obtained.

### Refinement

All H atoms were geometrically positioned (C—H 0.93–0.97 A*^o^*) and refined as riding, with *U*_iso_(H)= 1.2–1.5 *U*_eq_ of the parent atom.

### Crystal data

**Table:**

C_10_H_18_N_4_O_2_	*F*(000) = 244
*M**_r_* = 226.28	*D*_x_ = 1.264 Mg m^−^^3^
Monoclinic, *P*2_1_/*n*	Mo *K*α radiation, λ = 0.71073 Å
Hall symbol: -P 2yn	Cell parameters from 25 reflections
*a* = 4.4128 (3) Å	θ = 25–35°
*b* = 12.8534 (8) Å	µ = 0.09 mm^−^^1^
*c* = 10.4860 (7) Å	*T* = 293 K
β = 90.762 (2)°	Needle, colorless
*V* = 594.71 (7) Å^3^	0.16 × 0.13 × 0.12 mm
*Z* = 2	

### Data collection

**Table:**

Bruker SMART CCD area detector diffractometer	939 reflections with *I* > 2σ(*I*)
Radiation source: fine-focus sealed tube	*R*_int_ = 0.032
graphite	θ_max_ = 27.0°, θ_min_ = 2.5°
multi–scan	*h* = −5→5
3578 measured reflections	*k* = −16→16
1293 independent reflections	*l* = −13→7

### Refinement

**Table:**

Refinement on *F*^2^	Primary atom site location: structure-invariant direct methods
Least-squares matrix: full	Secondary atom site location: difference Fourier map
*R*[*F*^2^ > 2σ(*F*^2^)] = 0.049	Hydrogen site location: difference Fourier map
*wR*(*F*^2^) = 0.132	H-atom parameters constrained
*S* = 1.05	*w* = 1/[σ^2^(*F*_o_^2^) + (0.0571*P*)^2^ + 0.1569*P*] where *P* = (*F*_o_^2^ + 2*F*_c_^2^)/3
1293 reflections	(Δ/σ)_max_ < 0.001
75 parameters	Δρ_max_ = 0.16 e Å^−^^3^
0 restraints	Δρ_min_ = −0.22 e Å^−^^3^

### Special details

**Table:**

Geometry. All e.s.d.'s (except the e.s.d. in the dihedral angle between two l.s. planes) are estimated using the full covariance matrix. The cell e.s.d.'s are taken into account individually in the estimation of e.s.d.'s in distances, angles and torsion angles; correlations between e.s.d.'s in cell parameters are only used when they are defined by crystal symmetry. An approximate (isotropic) treatment of cell e.s.d.'s is used for estimating e.s.d.'s involving l.s. planes.
Refinement. Refinement of *F*^2^ against ALL reflections. The weighted *R*-factor *wR* and goodness of fit *S* are based on *F*^2^, conventional *R*-factors *R* are based on *F*, with *F* set to zero for negative *F*^2^. The threshold expression of *F*^2^ > σ(*F*^2^) is used only for calculating *R*-factors(gt) *etc*. and is not relevant to the choice of reflections for refinement. *R*-factors based on *F*^2^ are statistically about twice as large as those based on *F*, and *R*- factors based on ALL data will be even larger.

### Fractional atomic coordinates and isotropic or equivalent isotropic displacement parameters (Å^2^)

**Table:**

	*x*	*y*	*z*	*U*_iso_*/*U*_eq_	
O1	0.8167 (3)	0.17534 (10)	0.32669 (12)	0.0477 (4)	
H1	0.8704	0.1661	0.4010	0.072*	
N1	0.6414 (3)	0.26556 (11)	0.31833 (13)	0.0378 (4)	
N2	0.2568 (3)	0.40201 (11)	0.07864 (13)	0.0351 (4)	
C1	0.5506 (4)	0.28417 (13)	0.20383 (15)	0.0345 (4)	
C2	0.3631 (4)	0.37999 (13)	0.19019 (15)	0.0335 (4)	
C3	0.0664 (4)	0.49535 (14)	0.06676 (16)	0.0378 (4)	
H3A	0.1871	0.5566	0.0861	0.045*	
H3B	−0.0964	0.4918	0.1279	0.045*	
C4	0.6133 (5)	0.21689 (16)	0.09136 (17)	0.0521 (6)	
H4A	0.7442	0.2532	0.0340	0.078*	
H4B	0.4262	0.2003	0.0483	0.078*	
H4C	0.7100	0.1539	0.1194	0.078*	
C5	0.3088 (5)	0.44515 (16)	0.30710 (17)	0.0478 (5)	
H5A	0.3519	0.5167	0.2885	0.072*	
H5B	0.4387	0.4218	0.3754	0.072*	
H5C	0.1010	0.4385	0.3319	0.072*	

### Atomic displacement parameters (Å^2^)

**Table:**

	*U*^11^	*U*^22^	*U*^33^	*U*^12^	*U*^13^	*U*^23^
O1	0.0611 (9)	0.0471 (8)	0.0345 (7)	0.0125 (7)	−0.0118 (6)	0.0046 (6)
N1	0.0454 (9)	0.0382 (8)	0.0297 (8)	0.0030 (7)	−0.0085 (6)	0.0034 (6)
N2	0.0385 (8)	0.0369 (8)	0.0296 (7)	−0.0036 (6)	−0.0083 (6)	0.0039 (6)
C1	0.0380 (9)	0.0379 (9)	0.0274 (9)	−0.0049 (7)	−0.0057 (7)	0.0034 (7)
C2	0.0367 (9)	0.0367 (9)	0.0271 (8)	−0.0049 (7)	−0.0057 (7)	0.0022 (7)
C3	0.0404 (9)	0.0386 (10)	0.0343 (9)	−0.0016 (8)	−0.0083 (7)	0.0042 (7)
C4	0.0754 (15)	0.0487 (12)	0.0319 (10)	0.0139 (10)	−0.0097 (9)	−0.0010 (8)
C5	0.0622 (13)	0.0491 (11)	0.0319 (9)	0.0079 (9)	−0.0082 (8)	−0.0005 (8)

### Geometric parameters (Å, °)

**Table:**

O1—N1	1.3959 (19)	C3—H3A	0.9700
O1—H1	0.8200	C3—H3B	0.9700
N1—C1	1.283 (2)	C4—H4A	0.9600
N2—C2	1.286 (2)	C4—H4B	0.9600
N2—C3	1.469 (2)	C4—H4C	0.9600
C1—C2	1.490 (2)	C5—H5A	0.9600
C1—C4	1.491 (2)	C5—H5B	0.9600
C2—C5	1.507 (2)	C5—H5C	0.9600
C3—C3^i^	1.515 (3)		
			
N1—O1—H1	109.5	H3A—C3—H3B	108.1
C1—N1—O1	112.30 (14)	C1—C4—H4A	109.5
C2—N2—C3	117.27 (15)	C1—C4—H4B	109.5
N1—C1—C2	114.21 (15)	H4A—C4—H4B	109.5
N1—C1—C4	124.96 (16)	C1—C4—H4C	109.5
C2—C1—C4	120.81 (14)	H4A—C4—H4C	109.5
N2—C2—C1	117.70 (15)	H4B—C4—H4C	109.5
N2—C2—C5	123.89 (16)	C2—C5—H5A	109.5
C1—C2—C5	118.41 (14)	C2—C5—H5B	109.5
N2—C3—C3^i^	110.88 (19)	H5A—C5—H5B	109.5
N2—C3—H3A	109.5	C2—C5—H5C	109.5
C3^i^—C3—H3A	109.5	H5A—C5—H5C	109.5
N2—C3—H3B	109.5	H5B—C5—H5C	109.5
C3^i^—C3—H3B	109.5		
			
O1—N1—C1—C2	−179.86 (14)	C4—C1—C2—N2	0.4 (3)
O1—N1—C1—C4	1.8 (3)	N1—C1—C2—C5	1.9 (2)
C3—N2—C2—C1	178.63 (15)	C4—C1—C2—C5	−179.72 (18)
C3—N2—C2—C5	−1.3 (2)	C2—N2—C3—C3^i^	−174.31 (18)
N1—C1—C2—N2	−178.05 (16)		

Symmetry codes: (i) −*x*, −*y*+1, −*z*.

### Hydrogen-bond geometry (Å, °)

**Table:**

*D*—H···*A*	*D*—H	H···*A*	*D*···*A*	*D*—H···*A*
O1—H1···N2^ii^	0.82	2.12	2.8384 (19)	146

Symmetry codes: (ii) *x*+1/2, −*y*+1/2, *z*+1/2.
